# Case Report: Long Progression-Free Survival of Immunotherapy for Lung Adenocarcinoma With Epidermal Growth Factor Receptor Mutation

**DOI:** 10.3389/fonc.2021.731429

**Published:** 2021-09-23

**Authors:** Jianfeng Peng, Xianguang Zhao, Kaikai Zhao, Xiangjiao Meng

**Affiliations:** ^1^ Department of Radiation Oncology, Shandong Cancer Hospital Affiliated to Shandong First Medical University, Jinan, China; ^2^ Department of Radiation Oncology, Shandong Cancer Hospital, Jinan, China; ^3^ Department of Radiation Oncology, The First Affiliated Hospital of China Medical University, Shenyang, China; ^4^ Department of Radiation Oncology, Yantai Affiliated Hospital of Binzhou Medical University, Yantai, China

**Keywords:** non-small cell lung cancer (NSCLC), immunotherapy, EGFR mutation, EGFR-TKIs, biomarkers

## Abstract

**Background:**

Immune checkpoint inhibitors (ICIs) have been clinically proven to be efficient in non-small cell lung cancer (NSCLC). However, it has also been found that immunotherapy is not effective for all patients. For instance, some patients with epidermal growth factor receptor (EGFR) mutation tumors have a low overall response rate to ICIs. As a result, we retrospectively analyzed the efficacy of anti-programmed death-ligand 1 (anti-PD-L1) blockade (atezolizumab) treatment for a patient with EGFR mutation, and we explored the interaction between immunotherapy and EGFR mutations in NSCLC.

**Case Presentation:**

A patient, 62-year-old non-smoking female, with lung adenocarcinoma was initially misdiagnosed as EGFR wild type and received a third-line treatment with atezolizumab, experiencing partial response (PR) and progression-free survival (PFS) for 23 months. She had later been confirmed with EGFR L858R mutation prior to taking atezolizumab. On top of that, the patient developed T790M mutation after being administered with atezolizumab instead of EGFR tyrosine kinase inhibitors (TKIs). She started with osimertinib, although the lesion continued to progress. Tumor mutational burden (TMB), PD-L1 expression, and tumor-infiltrating lymphocytes (TILs) had been tested for further analysis.

**Conclusion:**

The case report and literature review indicate that ICIs might be more effective for L858R mutation than for other EGFR mutant subtypes, which correlates with certain potential predictors such as TMB and concurrent PD-L1 plus CD8^+^ TIL expression. However, there is no report on progression from non-primary EGFR T790M mutation to T790M mutation of patients who neither previously suffered from EGFR-TKIs nor responded to osimertinib. This case report will offer some information to guide the investigation on how to identify those who can benefit from immunotherapy and those who do not respond to EGFR-TKIs among the patients with EGFR mutations.

## Introduction

Immunotherapy using monoclonal antibodies directed at checkpoint proteins has become the newest treatment modality in lung cancer, including PD-1, programmed death-ligand 1 (PD-L1), and CTLA4 (1). Previous studies have shown better overall survival (OS) with immune checkpoint inhibitors (ICIs) compared with second- or third-line docetaxel (2). Compared with traditional cytotoxic chemotherapy, ICI monotherapy or combination therapy has shown to be a reliable and safe first- and second-line therapy for non-small cell lung cancer (NSCLC) without driver gene mutations (3–6). Thus, National Comprehensive Cancer Network (NCCN) guidelines recommends them as first-line and second-line treatments for NSCLC patients.

However, single-agent ICIs do not appear to have a striking advantage over cytotoxic agents in NSCLC harboring epidermal growth factor receptor (EGFR) mutations (2). As a major molecular subtype in Asian lung cancer patients, EGFR mutation accounts for 40% of lung adenocarcinoma, of which the two most common mutations are exon 19 deletion (60%) and L858R missense replacement (35%) (7). The specific criteria of the choice of EGFR tyrosine kinase inhibitors (TKIs) and ICIs for NSCLC patients with EGFR mutation appear to be the focus and challenge of the current research.

This case report describes a lung adenocarcinoma patient who achieved long progression-free survival (PFS) with an EGFR mutation treated with atezolizumab.

## Case Presentation

**Figure 1:**
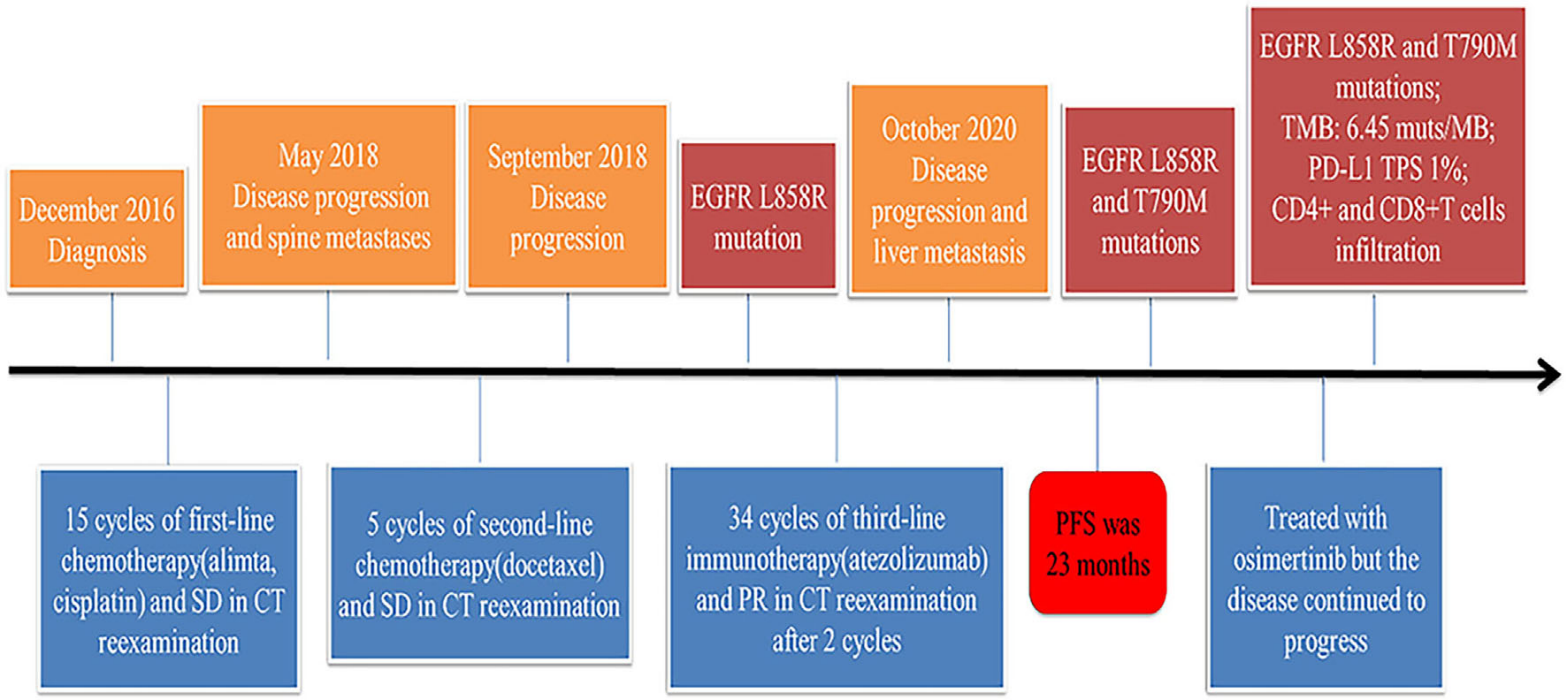
Timeline of disease status and corresponding treatment regimens.

A 62-year-old non-smoking woman with cough and chest tightness was initially found to have space-occupying lesions in her lungs in a physical examination at another hospital. Then she came to our hospital for further treatment. At this time, she had an Eastern Cooperative Oncology Group (ECOG) performance status of 1. And she had no relevant medical or surgical history. Enhanced CT showed a 3.0 × 3.0 cm mass in the left lower lung with diffuse miliary metastases in both lungs and no distant lymph node metastasis. The clinical stage was T1cN0M1a, and the pathological puncture diagnosis was lung adenocarcinoma in December 2016. She refused the gene test and received first-line treatment: pemetrexed 0.5 mg/m^2^ d1 + cisplatin 75 mg/m^2^ d1, q3w, for six cycles from January 2017 to June 2017. From July 2017 to April 2018, pemetrexed was given 0.8 d1, q3w, for nine cycles, and the best response was stable disease (SD) (**Figure 1**). In May 2018, CT reexamination revealed multiple vertebral metastases in the spine, and the efficacy was evaluated as progressive disease (PD). Therefore, second-line treatment regimen was used: docetaxel 120 mg d1, q21d, five cycles, from May 2018 to September 2018. Then CT reexamination after the end of treatment showed that the response was PD.

**Figure 2:**
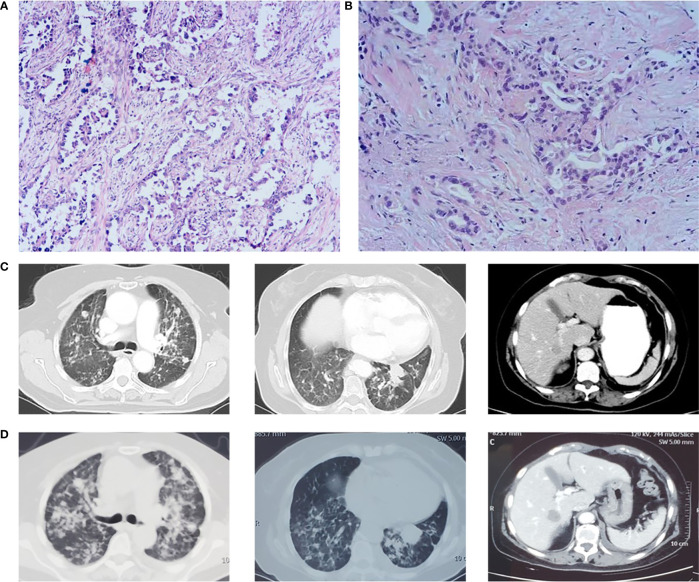
**(A)** Pathological biopsy on October 22, 2018. **(B)** Pathological biopsy on November 11, 2020. **(C)** Lung lesion progression and liver metastasis on October 13, 2020. **(D)** Lesion progression in March 2021 after osimertinib treatment.

**Figure 3:**
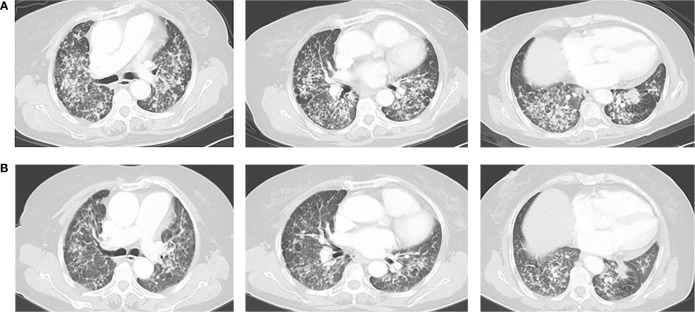
**(A)** Chest CT on October 25, 2018. **(B)** Chest CT on December 21, 2018. The response was partial response (PR).

This time, the patient expressed a strong desire for treatment, and we had full discussions with the patient and her family to determine the next step of treatment. On October 22, 2018, we performed genetic testing on the patient using PCR, and the results showed that EGFR, ALK, and ROS1 were all wild types (**Figure 2A**). Atezolizumab treatment was initiated on November 2, 2018, based on the results of the OAK clinical trial (6) and the guidelines. After two cycles, CT showed partial response (PR) (**Figure 3**).

At cycle 18 of atezolizumab, free triiodothyronine (FT3) decreased to 3.62 pmol/L, and the patient was treated with the addition of levothyroxine sodium tablet 50 μg qd po. Liver metastasis occurred, and lung disease progressed on October 13, 2020 (**Figure 2C**). PFS was as long as 23 months. The clinical stage at this time was T2aN0M1c. After discussion of the multidisciplinary team (MDT), pathological puncture and genetic testing were conducted again for the patient, and the results showed EGFR L858R and T790M mutations (**Figure 2B**). Research by Hsu et al. suggested that mutations in EGFR are associated with a higher rate of miliary lung metastases (8); for the sake of precision, we redid genetic testing on the patient’s 2018 pathological specimen using a more accurate next-generation sequencing (NGS) (9), and we found that the patient had the EGFR L858R mutation prior to the use of atezolizumab. Then the patient was treated with osimertinib in view of EGFR^T790M^, but the follow-up CT until March 2021 showed that the lesion continued to progress (**Figure 2D**).

**Figure 4:**
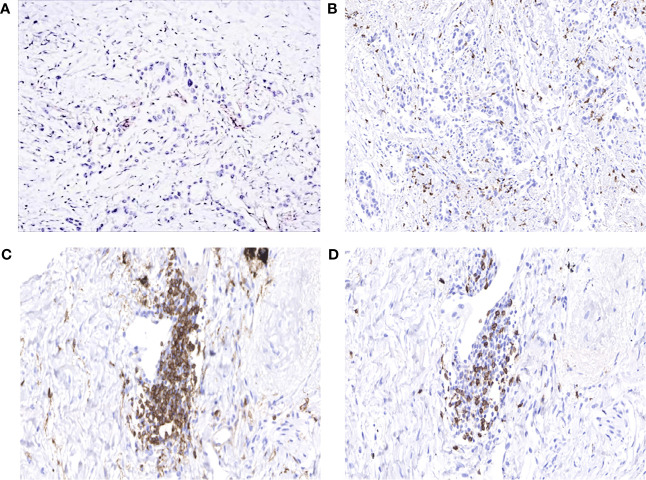
**(A)** Programmed death-ligand 1 (PD-L1) tumor proportion score (TPS) <1%. **(B)** High infiltration of CD8^+^ tumor-infiltrating lymphocytes (TILs). **(C)** Presence of large numbers of CD4^+^ T cells in the stroma of the tumor. **(D)** Presence of large numbers of CD8^+^ T cells in the stroma of the tumor.

In order to explore why the patient responded well to atezolizumab but not to osimertinib, we performed the whole gene test (825 gene) by NGS and immunohistochemistry (IHC). At this time, the patient carried EGFR L858R, T790M, and TP53 R282W mutations; and her plasma sample had a low tumor mutational burden (TMB) of 6.45 muts/Mb. IHC showed PD-L1 tumor proportion score (TPS) <1% (antibody was 22C3), but the expression of PD-L1 in immune cells was 5%. In addition, CD4^+^ and CD8^+^ T cells were highly infiltrated (**Figure 4**).

## Discussion

Atezolizumab, a kind of ICIs, is a humanized monoclonal antibody that can specifically bind to PD-L1 to alter the immune escape mechanism of tumors and plays a role in anti-tumor activity (10). Several clinical trials have shown that ICIs can significantly prolong PFS and OS in tumors, whereas the benefit is limited to patients with EGFR wild type and ALK wild type. The OAK study subgroup showed that the patients with EGFR mutation received similar OS benefit with atezolizumab and docetaxel (6), which is consistent with the results of the CheckMate 057 and Keynote 010 trials (5, 11).

In this case, the patient with EGFR L858R mutation who was not pretreated with EGFR-TKIs was treated with atezolizumab. She obtained an extremely long PFS, although previous studies show different results. Some clinical trials have shown that certain EGFR mutant subtypes of tumors do respond to ICIs; for example, Hastings et al. observed that patients with EGFR L858R mutation receiving immunotherapy showed similar response rates and OS as patients with EGFR wild-type lung cancer, but they had much worse PFS (12). Her longer PFS may be related to some biomarkers. The predictive biomarkers such as PD-L1 TPS in cancer cells and microsatellite instability (13) have been approved by the U.S. Food and Drug Administration (FDA) for determining suitable advanced NSCLC patients that can benefit from ICIs (13). Moreover, other markers, including TMB, tumor-infiltrating lymphocytes (TILs), and other components of tumor microenvironment (TME), are being actively investigated (13). High level of PD-L1 expression, TMB, and CD8^+^ TIL is associated with benefits of blocking PD-L1 and may lead to better PFS, OS, and response rates (13, 14). In this case, genetic testing and IHC of this patient showed low TMB level and low PD-L1 expression but a high level of CD8^+^ TILs, which is a beneficial advantage for patient receiving immunotherapy. CD8^+^ T cell can specifically recognize tumor antigens to kill tumors directly or target cells indirectly by secreting cytokines (15). In addition, previous studies have concluded that the expression of PD-L1 on both dendritic cells and macrophages is in relation to the efficacy of ICIs (16, 17). This patient’s PD-L1 expression in immune cells takes 5%, which might be one of the reasons for the response to atezolizumab. Furthermore, this patient was pretreated with multiline chemotherapeutic agents before receiving atezolizumab. Results from the ATLANTIC showed that when durvalumab is used as a third-line treatment or above, NSCLC patients who are with EGFR mutations and have at least 25% of tumor cells with PD-L1 expression could benefit from durvalumab with an objective response rate of 12.2% (9/74 patients, 95% CI 5.7–21.8). The objective response rate in patients accounting for less than 25% of tumor cells with PD-L1 expression is only 3.6% (1/28 patients, 95% CI 0.1–18.3) (18). Therefore, for this patient, further studies are needed to confirm whether NSCLC harboring EGFR L858R mutations with a high degree of CD8^+^ TILs might benefit from atezolizumab or even obtain longer PFS after having multiline chemotherapy. In addition, the whole gene testing of the patient revealed that she also carried the TP53 R282W mutation, which is considered to be a positive gene (19). Previous studies have found that TP53 mutation increases the expression of PD-L1, thus making patients more likely to benefit from immunotherapy (20). Wu and his team found that TP53 mutations not only triggered these changes but also increased the proportion of CD8^+^ T cells (21), which is consistent with this case. However, a clinical retrospective study has shown a contrasting result, suggesting that TP53 mutation is negatively correlated with immunotherapy efficacy (22). Therefore, subsequent studies are needed to confirm the correlation between TP53 mutation and immunotherapy and to clarify the relevant mechanisms.

Moreover, T790M mutations are a common resistance mutation, causing about 60% resistance to EGFR-TKIs (23). T790M mutations can be divided into two types—primary or acquired. Acquired T790M mutation is usually the resistant gene after the first or second generation of EGFR-TKIs, and both primary and secondary mutations have shown good responses to osimertinib (a type of third-generation EGFR-TKIs) therapy (24, 25). The patient presented in this report with non-primary EGFR T790M mutation who developed T790M mutation after ICIs rather than EGFR-TKIs, and she had a complete failure to respond to osimertinib. It has not been previously reported whether immunotherapy will induce new EGFR mutations or affect the efficacy of osimertinib. In addition, it is also worth exploring if high CD8^+^ TILs cause the above effect. A retrospective study conducted by Su et al. reports a high proportion of PD-L1^+^/CD8^+^ cases in advanced NSCLC patients who were *de novo* resistant to first-line EGFR-TKIs. These patients, despite their poor response to EGFR-TKIs, exhibited higher immunogenicity, which, as a result, may benefit from immunotherapy (26). Another retrospective study performed by Yoshiya et al. also found that EGFR-TKIs as first‐line treatment may have less benefit in EGFR-mutated tumors with both high expression of PD-L1 and CD8^+^ TILs (27). However, in their study, high CD8^+^ TIL (cohort 4) had longer PFS than low CD8^+^ TILs (cohort 2) under the same low expression level of PD-L1 can be observed (27). Due to the small sample sizes of these two groups, further studies are needed to be conducted to confirm the relationship between CD8^+^ TILs and the efficacy of EGFR-TKIs. In addition, it has been reported that the relative EGFR mutation abundance also could affect the therapeutic effect of using EGFR-TKI (28, 29). NGS of plasma performed prior to the application of osimertinib showed a mutation frequency of only 0.9% for EGFR T790M. The results of the study of Wang et al. showed that patients with low T790M mutation frequency are more likely to develop resistance and thus fail to benefit from osimertinib treatment (30).

However, there is one limitation to this case. Due to the insufficient pathological samples of patients saved before immunotherapy, we only performed genetic tests and could not perform IHC, so we did not know the levels of TMB, PD-L1 expression, and TILs before immunotherapy. For our subsequent studies, it is necessary to pay attention to the dynamic changes of biomarkers.

## Conclusion

At present, how to select immunotherapy regimens for NSCLC patients with EGFR mutations is still controversial, but it is undeniable that some mutant subtypes do respond to ICIs, which may be due to the different effects of EGFR mutation subtypes on PD-L1 expression, TMB, and TME, thus affecting the efficacy of ICIs. However, we do not know much about whether ICIs or CD8^+^ TILs affect the EGFR pathway and the efficacy of EGFR-TKIs. We need to further study this, so as to provide reference value for the selection of drug regimens for NSCLC with EGFR mutations in the future.

## Data Availability Statement

The datasets presented in this study can be found in online repositories. The names of the repository/repositories and accession number(s) can be found below: NCBI [accession: PRJNA752895].

## Ethics Statement

The studies involving human participants were reviewed and approved by Shan Dong Cancer Hospital and Institute. The patient provided her written informed consent to participate in this study. Written informed consent was obtained from the individual for the publication of any potentially identifiable images or data included in this article.

## Author Contributions

XM designed the study, edited, and approved the final manuscript. KZ and XZ collected clinical data and performed histological analysis. JP collected and analyzed the data and drafted the manuscript. All authors contributed to the article and approved the submitted version.

## Funding

This work was supported by the National Natural Science Foundation of China (grant numbers 81972796) and Natural Science Foundation of Shandong Province (grant numbers ZR2019MH010 and ZR2019MH289).

## Conflict of Interest

The authors declare that the research was conducted in the absence of any commercial or financial relationships that could be construed as a potential conflict of interest.

## Publisher’s Note

All claims expressed in this article are solely those of the authors and do not necessarily represent those of their affiliated organizations, or those of the publisher, the editors and the reviewers. Any product that may be evaluated in this article, or claim that may be made by its manufacturer, is not guaranteed or endorsed by the publisher.
